# ZBP1 as a dynamic monitor of viral replication: implications for therapeutic strategies

**DOI:** 10.3389/fcimb.2026.1758221

**Published:** 2026-02-09

**Authors:** Zhiying Ou, Yanfeng Huang, Xi Xue, Huiling Zhou, Shuihong Li, Kangpeng Xiao

**Affiliations:** 1 Institute of Pathogenic Biology, College of Basic Medical Sciences, Hengyang Medical School, University of South China, Hengyang, China; 2 MOE Key Lab of Rare Pediatric Diseases, Hengyang Medical School, University of South China, Hengyang, China

**Keywords:** immune evasion, PANoptosis, spatiotemporal dynamics, viral replication, ZBP1, Z-NAs

## Abstract

Z-DNA binding protein 1 (ZBP1) is an innate immune sensor that recognizes Z-NAs, an atypical, left-handed nucleic acid structure produced during viral replication. This review contextualizes ZBP1 function within the spatiotemporal dynamics of the viral replication cycle, portraying it as a dynamic monitor rather than a static alarm. We discuss how the subcellular localization determines the signaling outcome (e.g., nuclear versus cytoplasmic sensing). Specifically, we discuss how ZBP1 functions as a dynamic molecular scaffold, where ligand-induced amyloid assembly concentrates downstream kinases to overcome cellular inhibition and initiate cell death. The review details ZBP1’s dual antiviral strategy, encompassing NF-κB-mediated inflammation and PANoptotic cell death, and the resulting co-evolutionary dynamics, characterized by viral countermeasures such as ‘signal masking’ seen in poxvirus E3 and ‘signal interception’ utilized by herpesvirus ICP6. Finally, the dual immunomodulatory role of ZBP1 in driving immunopathology is analyzed. This replication-centric perspective provides a theoretical foundation for developing precise, stage-based therapies targeting the ZBP1 pathway.

## 1 Introduction

As obligate intracellular pathogens, viruses depend entirely on the host cell’s biosynthetic machinery for their life cycle. A typical viral replication cycle involves several stages, including attachment, entry, uncoating, biosynthesis, assembly, and release (Jones et al., 2021). Among these, biosynthesis, defined as the replication of the viral genome and synthesis of viral proteins, is not only the core of viral proliferation but also the stage of most intense host-virus interaction (Iselin et al., 2022). During replication, viruses inevitably produce molecular structures rare in host cells, particularly aberrant nucleic acid forms. These are termed Pathogen-Associated Molecular Patterns (PAMPs) (Malone et al., 2022), which act as critical signals for the host innate immune system to recognize non-self invasion and trigger antiviral defenses (Ren et al., 2020). Therefore, viral replication is a process that continuously exposes the virus, providing clues for immune surveillance.

To counter viral invasion, host cells have evolved Pattern Recognition Receptors (PRRs) to detect replication-associated PAMPs (Li and Wu, 2021). Among these, Z-DNA binding protein 1 (ZBP1), an Interferon-Stimulated Gene (ISG), has garnered widespread attention (Maelfait and Rehwinkel, 2023). ZBP1 is unique in its ability to recognize and bind the left-handed helical conformation of “Z-type” nucleic acids (Z-NAs). As detailed in this review, these Z-NAs are not merely static viral products; they accumulate dynamically from both viral replication intermediates and virus-induced host transcriptional stress, serving as a cumulative gauge of replication intensity (Yin et al., 2025). Significantly, ZBP1 also acts as a downstream effector for other innate pathways; for example, activation of the cGAS-STING DNA sensing pathway induces ZBP1-mediated necroptosis independently of canonical effectors like TNFR1 and FADD, revealing important crosstalk between these systems (Kelepouras et al., 2025). This capacity for precise recognition of a specific three-dimensional conformation makes ZBP1 a dedicated sensor for monitoring the dynamics of viral replication, as it recognizes not the virus itself, but the signature of its active replication.

Although ZBP1’s role as a Z-NAs sensor is well-established, its function is dynamic. ZBP1 activation, its effector functions, and tactics for viral evasion are all closely related to the stages, locations, and products of the viral replication cycle (Nagata et al., 2024). This review focuses on the inherent link between ZBP1’s immune function and the spatiotemporal dynamics of viral replication, examining their co evolution and interaction. Two significant aspects make this relationship apparent. First, ZBP1 activation is not just spatially matched but dictates mechanistically distinct outcomes. For instance, Influenza A Virus (IAV) sensing in the nucleus triggers hyperinflammatory nuclear necroptosis (Hao et al., 2022), a qualitatively different event from cytoplasmic activation (Balachandran and Mocarski, 2021). Second, this efficient monitoring applies strong selective pressure on viruses, compelling them to develop distinct antagonistic mechanisms (Maelfait and Rehwinkel, 2023).

By synthesizing and appraising these dynamic relationships, the work hopes to present a new virus replication focused view on ZBP1 function. This review synthesizes current insights into the interaction between ZBP1 surveillance and viral replication. Specifically, it elucidates how ZBP1 integrates spatiotemporal cues, including physical activation thresholds, isoform-mediated regulation, and the functional separation of signaling outcomes, to orchestrate antiviral defense. In parallel, the distinct mechanisms viruses have evolved to counteract these specific regulatory steps are analyzed. Finally, the discussion addresses the clinical implications of this host-virus interplay, highlighting emerging strategies for targeted therapeutic intervention.

## 2 Spatiotemporal dynamics and activation thresholds in Z-NAs recognition

### 2.1 Nuclear versus cytoplasmic Z-NAs sensing and its immunological implications

**Figure 1:**
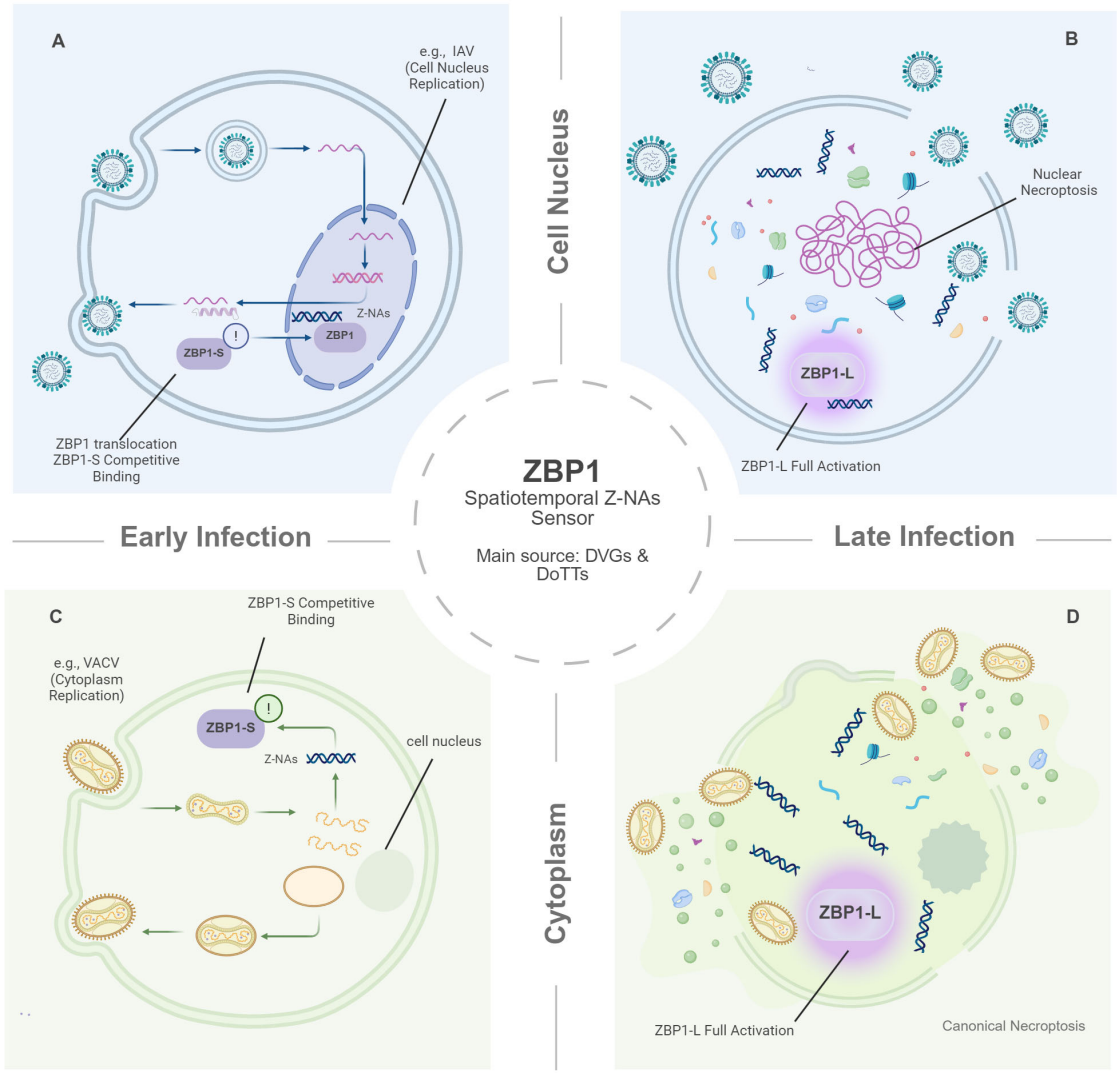
Schematic model of the spatiotemporal dynamics and threshold-dependent activation of ZBP1. The diagram illustrates ZBP1 as a dynamic sensor detecting Z-NAs (primarily derived from DVGs and DoTTs), whose activation is strictly governed by the subcellular location (Nucleus vs. Cytoplasm) and the stage of viral replication (Early vs. Late). **(A, B)** Nuclear Sensing (e.g., IAV). **(A)** Early Infection: ZBP1 translocates from the cytoplasm to the nucleus. In this model, low levels of nascent Z-NAs are competitively bound by the inhibitory isoform ZBP1-S, keeping ZBP1 below the activation threshold and triggering only an initial alert. **(B)** Late Infection: According to the proposed DoTT model, transcription generates massive accumulation of Z-NAs, which is proposed to overwhelm ZBP1-S. This surplus clusters the full-length isoform (ZBP1-L), driving full activation and “nuclear necroptosis” (nuclear rupture). **(C, D)** Cytoplasmic Sensing (e.g., Poxviruses). **(C)** Early Infection: Viral replication initiates in the cytoplasm. Low-abundance Z-NAs are similarly sequestered by ZBP1-S competitive binding, preventing premature cell death. **(D)** Late Infection: Massive genome amplification increases cytoplasmic Z-RNA density. This breaches the biophysical threshold, leading to ZBP1-L full activation and canonical cell death (e.g., PANoptosis) to destroy the viral replication compartment. Created with BioRender.com.

The subcellular location of viral replication largely defines the site of Z-NAs production. This spatial context typically influences whether ZBP1 sensing occurs in the nucleus or the cytoplasm (Ingram et al., 2019). Using IAV as a model, recent work illustrates how this spatial distinction drives distinct cell death modalities. IAV recruits ZBP1 to the nucleus via viral ribonucleoproteins (vRNPs) (**Figure 1A**). Here, nuclear sensing triggers “nuclear necroptosis” characterized by nuclear envelope rupture prior to plasma membrane lysis (**Figure 1B**) (Zhang et al., 2020). Crucially, this nuclear breakdown drives a hyper-inflammatory profile by releasing nuclear DAMPs (e.g., IL-33, HMGB1) (Gautam et al., 2024). This unique topography is immunologically decisive. In sharp contrast, poxviruses like Mpox virus and vaccinia virus (VACV) replicate entirely in the cytoplasm (Park and Walsh, 2022). In these cytoplasmic contexts, ZBP1 activation triggers canonical necroptosis primarily targeting the plasma membrane without the distinct nuclear envelope breakdown observed in IAV (**Figures 1C, D**). Even within the nucleus, outcomes vary. While IAV drives nuclear rupture, other nuclear viruses like herpesviruses typically lack this phenotype (Guo et al., 2015). However, the precise reason for this absence remains unclear. The presence of antagonists like ICP6 (Shanmugam et al., 2021), which intercept signaling, makes it difficult to determine whether these viruses naturally lack the capacity for nuclear rupture or if the phenotype is simply masked by viral inhibition. Consequently, comparing these viral classes underscores a critical insight that ZBP1 sensing is a plastic mechanism, governed by the specific interplay between viral localization and immune evasion strategies.

### 2.2 Origins of Z-NAs from viral byproducts and virus-induced host transcriptional stress

The Z-NAs that activate ZBP1 arise from multiple sources that include viral replication byproducts and virus-induced abnormalities in host transcription. Defective viral genomes (DVGs), truncated species, and palindromic sequences can fold into local double-stranded RNA and adopt the Z conformation, as observed in infections with IAV and Reovirus (Guo et al., 2018; DeAntoneo et al., 2022; He et al., 2025). Nevertheless, these species do not fully explain ZBP1 activation in all contexts, since robust ZBP1 activation is observed even during influenza infection with a low burden of DVGs. Building on this, recent work proposes a model termed Disruption of Transcriptional Termination (DoTT), primarily characterized during IAV infection, where viral interference with host RNA processing machinery generates large numbers of host transcripts with excessively long 3’ extensions (**Figure 1**) (Yin et al., 2025). These extensions are enriched for endogenous retroelements, specifically SINEs and LINEs. Their inverted repeats promote intramolecular pairing to form stable dsRNA that has the potential to adopt Z conformations, which in turn represents a potential source of ZBP1 ligands (Nacken et al., 2021; Yin et al., 2025). Taken together, these observations suggest that ZBP1 may sense not only products of active, high-intensity viral replication but also virus-driven damage to the host transcriptional machinery, positioning it as a sensitive indicator of pathological transcriptional stress in infected cells. At the same time, detecting Z-NAs *in situ* remains challenging because the Z conformation is intrinsically labile and can be shielded by viral or host proteins, potentially leading to underestimation of its true abundance (Yin et al., 2023). Future studies should develop more sensitive enrichment, sequencing, and spatial mapping strategies to resolve the spatiotemporal dynamics of Z-NAs during infection with greater precision.

### 2.3 ZBP1 as a molecular scaffold for PANoptosis and inflammatory signaling

Viral replication dynamics suggest fluctuating Z-RNA abundance across infection stages (Mishra et al., 2024). While direct quantification remains challenging, biophysical and structural evidence may involve a threshold-like behavior. At the molecular level, ZBP1 activation depends on higher-order assembly (Peng et al., 2022). Viral Z-NAs recognition by its N-terminal Zα domains triggers a conformational shift exposing the RIP homotypic interaction motif (RHIM) (Xie et al., 2024), enabling homotypic interactions with RIPK3 and RIPK1 (Koerner et al., 2024). This contact drives the rapid oligomerization of these proteins into amyloid-like signaling complexes (Polonio et al., 2025). Within this dense architecture, the signaling components are brought into close physical proximity. This organization may facilitate proximity-driven kinase activation and help overcome inhibitory constraints (Koerner et al., 2024; Amusan et al., 2025). Specifically, recent studies identify an inhibitory splice isoform (ZBP1-S) lacking RHIM domains as a key modulator of this threshold. ZBP1-S competitively binds Z-NAs and constrains oligomerization of the full-length isoform (ZBP1-L), thereby modulating the ligand threshold required for robust pathway activation (**Figure 1**) (Cai et al., 2024; Nagata et al., 2024). Thus, this RHIM-dependent core functions as a central signaling hub to orchestrate convergent PANoptosis and inflammatory cascades. Given the mechanistic complexity and viral diversity, we distinguish established evidence from emerging conceptual models in **Table 1**.

**Table 1:** Summary of established evidence and emerging working models in ZBP1 signaling.

Key topic	Established experimental evidence	Emerging conceptual models
Generation of Z-NAs	ZBP1 detects Z-NAs directly from viral genomes including defective viral genomes and replication intermediates (Guo et al., 2018; Zhang et al., 2020).	ZBP1 may sense host transcriptional stress via endogenous retroelements accumulated during DoTT (Yin et al., 2025).
Mechanism of Activation	ZBP1-L binds Z-NAs to recruit RIPK1/3; ZBP1-S acts as a competitive inhibitor of this pathway (Maelfait and Rehwinkel, 2023; Nagata et al., 2024).	ZBP1-S may set a threshold breached in late infection, potentially via higher-order assemblies (Cai et al., 2024; Xie et al., 2024; Amusan et al., 2025).
Spatiotemporal Regulation	The distinct nuclear or cytoplasmic location of viral replication can shape which ZBP1 pool is engaged (Balachandran and Mocarski, 2021).	Nuclear sensing has been associated with nuclear necroptosis characterized by envelope rupture in IAV but its generality across viruses remains uncertain (Zhang et al., 2020; Gautam et al., 2024).

## 3 Antiviral effector functions spanning infected cell clearance and immune potentiation

### 3.1 PANoptosis as a convergent cell death pathway for infected cell clearance

**Figure 2:**
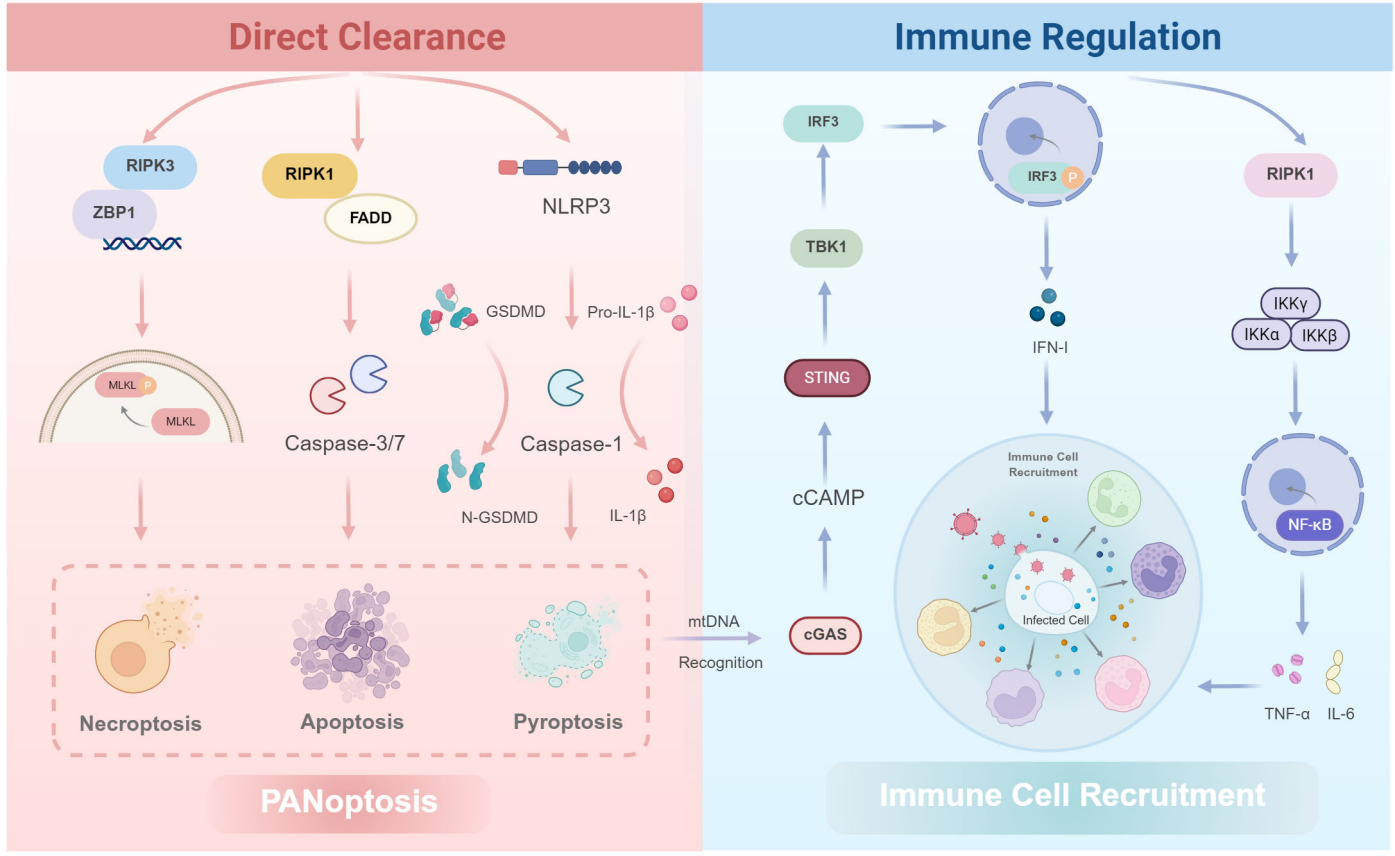
The dual effector functions of ZBP1 in antiviral defense: direct clearance via PANoptosis and broad immune regulation. Upon sensing Z-NAS, ZBP1 initiates two distinct sets of pathways. (Left Panel: Direct Clearance) ZBP1 triggers PANoptosis, a composite cell death program, by recruiting RIPK3 to initiate MLKL-driven necroptosis. This platform also recruits RIPK1 and FADD to activate Caspase-8-mediated apoptosis. Furthermore, Caspase-8 activation promotes the assembly of the NLRP3 inflammasome, leading to Caspase-1-driven pyroptosis, characterized by GSDMD cleavage and IL-1β maturation. This multi-arm redundancy ensures the termination of viral replication. (Right Panel: Immune Regulation) Independent of cell death, ZBP1 utilizes RIPK1 to activate the IKK-NF-κB axis, inducing pro-inflammatory cytokines (e.g., TNF-α, IL-6). Concurrently, ZBP1-driven necroptosis releases mitochondrial DNA (mtDNA), which is sensed by cGAS, activating the STING-TBK1-IRF3 pathway to amplify Type I Interferon (IFN-I) production. Collectively, these actions clear the infected cell and establish a robust antiviral state by recruiting other immune cells. Created with BioRender.com.

A fundamental component of the antiviral strategies driven by ZBP1 is the induction of PANoptosis (Malireddi et al., 2023). This integrated cell-death paradigm eliminates infected host cells to halt viral spread (**Figure 2**). Conceptually, the essence of this integrated process lies in its parallel, multi-arm architecture, which ensures that even when viruses evolve inhibitors against a single pathway, the host can still clear infection through alternative routes. Mechanistically, ZBP1 engages its RHIM domain to recruit and activate RIPK3, driving necroptosis via MLKL phosphorylation and plasma membrane rupture (Koerner et al., 2024; Amusan et al., 2025). In parallel, the ZBP1–RIPK3 platform functions as a signaling scaffold that recruits RIPK1 and the adaptor FADD, thereby leading to activation of the initiator caspase-8. Once activated, caspase-8 not only cleaves and activates the executioner caspases-3 and -7 to trigger classical apoptosis (Rodriguez et al., 2022), but also acts as a critical node of cross-talk that promotes assembly of the NLRP3 inflammasome, resulting in caspase-1 activation (Malireddi et al., 2023). Consequently, caspase-1 performs dual effector functions, including the cleavage of gasdermin D (GSDMD) to generate membrane pores and drive pyroptosis, and it processes pro-IL-1β and pro-IL-18 into their mature, secreted forms. This multi-pathway design provides substantial defense redundancy (Evavold et al., 2021; Dong et al., 2024). Therefore, even in the face of numerous viral caspase inhibitors and other immune-evasion proteins, the pathway ensures convergence on cell death, such that at least one downstream pathway remains functionally active. Ultimately, this strategy represents an advanced host countermeasure to viral immune evasion, sacrificing individual infected cells to prevent organism-wide dissemination.

### 3.2 NF-κB activation and cGAS-STING synergy in immune signaling

Beyond its role in cell death, ZBP1 orchestrates a robust, cell-death-independent immune response composed of two key arms. First, it rapidly induces NF-κB dependent inflammation (Li et al., 2024a). Second, it cooperates with the cGAS-STING pathway to amplify type I interferon (IFN-I) production (Gomes et al., 2024). Together, these coordinated actions create a powerful antiviral state (**Figure 2**). Mechanistically, ZBP1 efficiently engages NF-κB signaling through its RHIM domain. By recruiting RIPK1 to assemble a signaling platform that activates the IKK complex, resulting in IκBα degradation and consequent nuclear translocation of NF-κB. Once in the nucleus, NF-κB promptly induces a wide array of pro-inflammatory genes, including TNF-α and IL-6, creating a local inflammatory milieu that functions as an immediate immune response at sites of infection (Imai et al., 2024; Koerner et al., 2024). Concurrently, functional synergy between ZBP1 and the cGAS-STING axis serves as a principal amplifier. This is initiated when ZBP1-driven necroptosis promotes the release of mitochondrial and other endogenous DNA into the cytosol, which is then sensed by cGAS to activate STING. This activation, occurring via the TBK1-IRF3 axis, robustly increases IFN-I production. Subsequently, the secreted IFN-I functions synergistically with pro-inflammatory cytokines, such as TNF-α and IL-6, produced by the NF-κB pathway (Yang et al., 2021; Ding et al., 2025). Thus, by functioning as an upstream apical regulator, ZBP1 tightly integrates rapid, localized inflammation with a potent systemic interferon response, promoting a comprehensive antiviral state essential for the efficient clearance of viral replication.

## 4 Molecular strategies for viral antagonism of ZBP1 surveillance

### 4.1 Ligand minimization through replication fidelity and RNA remodeling

**Figure 3:**
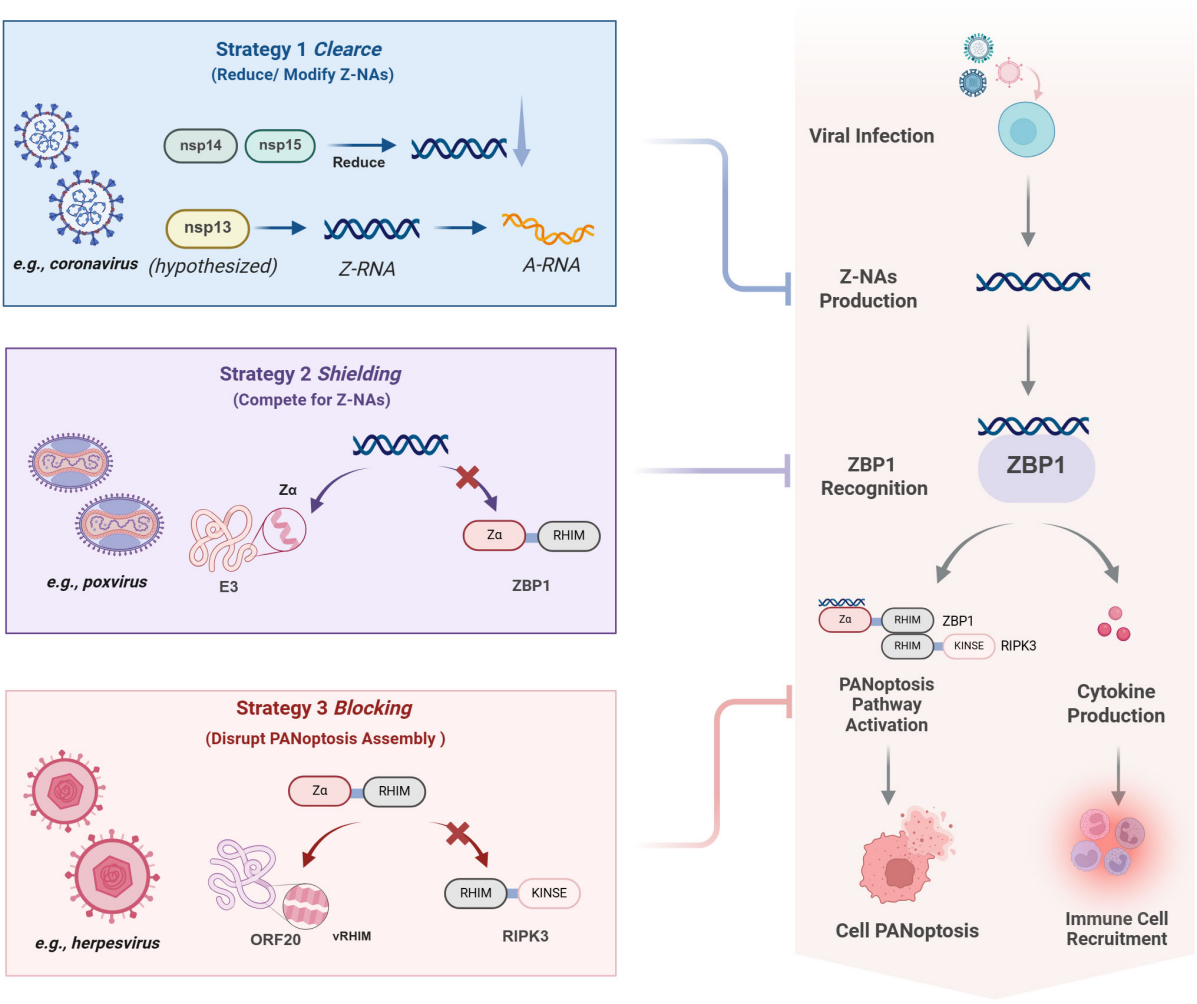
Three strategies of viral evasion of ZBP1 sensing. The figure illustrates viral interference at three key stages of the canonical ZBP1 antiviral pathway. (1) Signal Prevention: A proposed stealth strategy where some viruses (e.g., Coronaviruses) reduce Z-NAs production or reverse its conformation at the source via proteins like nsp14, nsp15, and nsp13 (hypothesized), preventing ZBP1 sensing. (2) Signal Shielding: Some viral proteins (e.g., Poxvirus E3) use their Zα domain to competitively bind Z-NAS, blocking ZBP1 recognition. (3) Signal Blocking: Other viral proteins (e.g., Herpesvirus vRHIMs) act as molecular mimics to disrupt the ZBP1-RIPK3 complex assembly, cutting off downstream cell death signals. Red “X” symbols indicate steps inhibited by the virus. Created with BioRender.com.

A fundamental mechanism limiting ligand generation (Strategy 1) involves the utilization of replication strategies that intrinsically minimize Z-NAs formation, thereby reducing the likelihood of detection by the ZBP1 surveillance system(**Figure 3**). The formation of Z-NAs is closely associated with imperfect replication processes, such as defective viral genomes (DVGs) produced by low-fidelity RNA polymerases or complementary RNA strands arising from overlapping transcriptional units (Brennan and Sun, 2024). Accordingly, any viral mechanism that enhances replication fidelity or reduces dsRNA by-products may indirectly facilitate evasion of ZBP1 (Gribble et al., 2021; Dupre and Volmer, 2023). Although there is currently no direct evidence that selection pressure from ZBP1 has driven the evolution of polymerase fidelity, certain virus-encoded proteins do have the potential to diminish immunostimulatory RNA. For instance, coronaviruses ubiquitously encode conserved enzymes that minimize immunostimulatory byproducts. Specifically, the exonuclease nsp14 confers proofreading capacity to reduce defective viral genomes (Ogando et al., 2020), while the endoribonuclease nsp15 cleaves viral RNA intermediates (Zhang et al., 2023a). Together, these enzymatic activities limit the accumulation of immunostimulatory RNA during replication (Gribble et al., 2021; Otter et al., 2024), thereby reducing the potential for Z-NA formation and subsequent detection by ZBP1 (Yang et al., 2025b). While these mechanisms likely evolved primarily for genomic stability rather than specifically to evade ZBP1, they objectively reduce the likelihood of ZBP1 activation. Furthermore, a computational study hypothesized that SARS-CoV-2 nsp13 might convert Z-RNA to A-RNA through its helicase activity, although this remains experimentally unvalidated, thereby directly erasing the ligand for ZBP1 (Herbert and Poptsova, 2022). Notably, future experimental validation of nsp13’s potential Z-RNA disruption could reveal novel strategies of immune avoidance that physically destabilize PAMP molecules, potentially offering a new perspective on how viruses modulate host innate immunity.

### 4.2 Ligand sequestration by viral Zα domains as molecular decoys

However, when rapid viral replication makes the generation of Z-NAs unavoidable, viruses must deploy a distinct evasion strategy (Strategy 2) to sequester these ligands (**Figure 3**). Some viruses, particularly poxviruses, have evolved a “signal-masking” strategy whereby, during the early phase of viral replication, proteins bearing a Zα domain are expressed to competitively bind and conceal newly produced viral Z-NAs (Koehler et al., 2021). Classic examples include Mpox virus (Li et al., 2023b) and VACV (Hsu et al., 2024), which both encode the multifunctional E3 protein containing an N-terminal Zα domain that structurally mimics host ZBP1 to competitively bind Z-NAs (Park et al., 2020). Crucially, as an early-gene product expressed shortly after viral entry, E3 acts as a pre-emptive shield that sequesters Z-NAs immediately upon their generation, thereby blocking ZBP1 recognition at the source (Soday et al., 2019; Pham et al., 2023). While intact E3 effectively suppresses ZBP1-dependent necroptosis by preventing ligand access (Zhang et al., 2023b), its mutation or deletion triggers robust cell death, resulting in impaired replication and markedly reduced virulence (Wang et al., 2024). This creates the necessary conditions for subsequent genome replication, late-stage protein synthesis, and the assembly of progeny virions. Therefore, the Zα domain of E3 is a key determinant of successful poxviral replication, achieving effective shielding from host immune surveillance by directly competing with ZBP1 for the ligand at the molecular level.

### 4.3 Signal disruption via viral RHIM mimics impairing necrosome assembly

Distinct from ligand sequestration, herpesviruses employ a strategy (Strategy 3) that targets downstream signaling rather than the ligand itself (**Figure 3**). Members of the subfamily Alphaherpesvirinae exemplify this strategy. For example, similar to the well-characterized HSV-1, pseudorabies virus (PRV) (Lyu et al., 2020) and equine herpesvirus 1 (EHV-1) (Vereecke et al., 2021) each encode viral RHIM-containing proteins (vRHIMs) homologous to ICP6. These early-gene products function as molecular mimics, interacting with the RHIM domains of ZBP1 or RIPK3 through homotypic binding (Shanmugam et al., 2021; Lodha et al., 2023). Consequently, such interactions exert a competitively inhibitory effect that disrupts proper assembly of the host ZBP1-RIPK3 signaling complex, preventing effective activation of downstream MLKL and thus blocking execution of necroptosis (Petrie et al., 2019). Similarly, a related mechanism has been identified in varicella-zoster virus (VZV), whose ORF20 protein also contains a RHIM domain and has been shown to inhibit ZBP1-dependent necroptosis (Steain et al., 2020; Verdonck et al., 2022). The evolutionary advantage of this approach likely stems from the multifunctional nature of vRHIMs, which integrate immune evasion functions into essential viral proteins (Ng et al., 2022). In this way, through molecular mimicry, herpesviruses specifically disrupt the integrity of the ZBP1 signaling axis. This ensures that even upon ZBP1 activation, host cells fail to execute cell death, thereby preserving a stable intracellular environment for viral genome replication and virion maturation (Wu et al., 2023).

## 5 Context-dependent modulation of the ZBP1 pathway for therapeutic intervention

### 5.1 Strategies re-activating ZBP1 signaling to counter viral antagonism

**Figure 4:**
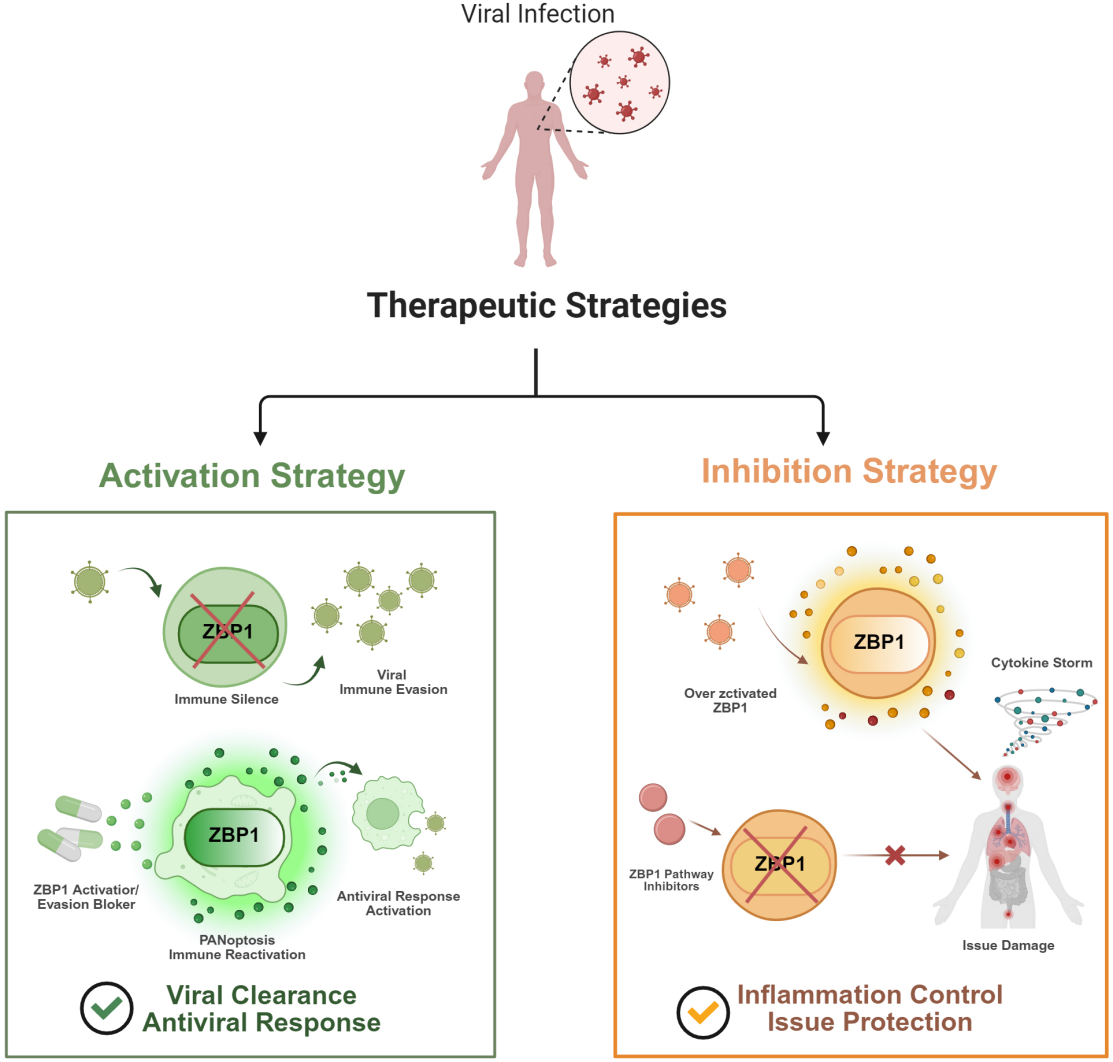
Dichotomous therapeutic strategies targeting the ZBP1 pathway in viral infection. Therapeutic interventions targeting ZBP1 must be tailored to the specific pathological context of the viral infection. (Left) Activation Strategy: In scenarios of viral immune evasion, viruses inhibit ZBP1 to minimize immune detection. Therapeutic agonists are deployed to override this blockade, reactivating ZBP1 to trigger PANoptosis. This restores the antiviral response and promotes the clearance of infected cells. (Right) Inhibition Strategy: Conversely, in conditions driven by hyperinflammation (e.g., cytokine storms in severe COVID-19), ZBP1 overactivation contributes to tissue injury. In these cases, pathway inhibitors are used to dampen ZBP1 signaling, preventing excessive damage and protecting organ function. This framework illustrates the opposing pathological roles of ZBP1, emphasizing the need for precise, context-dependent modulation. Created with BioRender.com.

For viruses employing antagonists to evade surveillance, re-activating ZBP1 represents a rational strategy to abort replication and restore host defense (**Figure 4**). Because direct ZBP1 agonists are currently unavailable, most translational strategies rely on repurposing existing drugs to activate the pathway indirectly. One widely used upstream approach is IFN-I therapy. As ZBP1 is an interferon-stimulated gene, IFN-α/β treatment markedly increases its expression (Yang et al., 2020), thereby lowering the threshold required for ZBP1 activation during viral infection, including settings where viral proteins antagonize upstream sensing (Koehler et al., 2017; Oh and Lee, 2023). Another strategy focuses on increasing endogenous ligands that can activate ZBP1. DNA hypomethylating agents, such as 5-azacytidine, can reactivate endogenous retroviruses and lead to the accumulation of cytosolic dsRNA (Noronha et al., 2024). In addition, the curaxin CBL0137 promotes the formation of Z-conformation DNA from chromatin (Wang et al., 2021; Li et al., 2024b). These nucleic acid structures can then be detected by ZBP1. Sensitization of downstream signaling has also been explored using SMAC mimetics, including birinapant. These compounds induce the degradation of cIAP1/2, thereby removing inhibitory constraints on the RIPK1–RIPK3 axis. As a result, necroptosis is more readily triggered, particularly when caspase-8 function is impaired (Ebert et al., 2015; Brumatti et al., 2016). Beyond host-directed approaches, targeting viral antagonists represents another possible direction. For example, future therapies could aim to disrupt the interaction between viral proteins such as vaccinia virus E3 and Z-NAs (Koehler et al., 2021). Although such interactions may be difficult to target structurally, interfering with viral suppression of ZBP1 could offer a potential strategy to re-engage the host’s antiviral surveillance. While ZBP1 activation strategies may benefit infections with potent viral antagonists, their utility against viruses with minimal evasion mechanisms remains uncertain, as excessive pathway engagement risks immunopathology (Li et al., 2023a; Boyd et al., 2025).

### 5.2 Established interventions targeting ZBP1-driven immunopathology

In many severe viral infections, such as severe COVID-19 or influenza, ZBP1 hyperactivation is a primary driver of lethal immunopathology and cytokine storms (Liang et al., 2024). Therefore, modulating ZBP1 signaling to avoid “auto-destructive” immune injury is a key and data-supported clinical goal. One clinically approved strategy targets ZBP1 expression (**Figure 4**). ZBP1 is a well-established Interferon-Stimulated Gene (ISG) (Yang et al., 2020). In severe COVID-19, ZBP1-driven inflammatory cell death creates a pathological feedback loop (Li et al., 2023a). The JAK1/JAK2 inhibitor Baricitinib, approved for severe COVID-19, is pertinent to this pathway. Its mechanism involves inhibiting JAK/STAT signaling, thereby suppressing the interferon-driven expression of ISGs, including ZBP1 (Kalil et al., 2021). A second strategy, which has advanced into human clinical trials, targets the downstream kinase RIPK1. Advanced, brain-penetrant RIPK1 inhibitors such as DNL104 and SAR443060 (DNL747) have completed Phase I and Phase Ib clinical trials for human neuroinflammatory diseases, including ALS and Alzheimer’s (Vissers et al., 2022). Finally, specific evidence demonstrates that the downstream MLKL-executioner arm of necroptosis can be pharmacologically targeted in viral infection models. The selective RIPK3 inhibitor UH15–38 effectively blocks IAV-induced necroptosis by inhibiting RIPK3 activation and subsequent MLKL phosphorylation (Gautam et al., 2024). Furthermore, *in vivo* studies with Severe Fever with Thrombocytopenia Syndrome Virus (SFTSV) demonstrated that pharmacological inhibition of MLKL decreased fatality (Li et al., 2025). The aforementioned strategies are indirect, focusing on upstream expression or downstream effectors. In contrast, direct targeting of ZBP1 has been difficult, mainly because the protein does not contain clear druggable binding pockets. More recently, covalent PROTACs have been developed to selectively degrade ZBP1 (Huang et al., 2025). These bifunctional molecules recruit an E3 ubiquitin ligase to ZBP1, leading to its ubiquitination and proteasomal degradation. Collectively, the integration of established upstream suppression, downstream blockade, and emerging direct degradation strategies provides a comprehensive framework for clinical intervention. This multi-level approach allows for the precise management of ZBP1-driven immunopathology.

## 6 Discussion

As detailed throughout this review, ZBP1 is essential for antiviral immunity, but it does not behave as a simple static on–off module. Instead, ZBP1 functions as a dynamic surveillance system closely tied to the viral replication cycle. In terms of spatial regulation, ZBP1 activation appears to be shaped by its subcellular localization rather than being determined by a single fixed outcome. Spatially, the divergent outcomes of nuclear versus cytoplasmic sensing underscore the plasticity of ZBP1 signaling, suggesting that the sensor’s location shapes the qualitative nature of the immune response, not just its initiation (Gautam et al., 2024). At the molecular level, ZBP1 recognizes Z-NAs, a potential marker of replication disruption associated with viral byproducts or host retroelements (DoTT) (Yin et al., 2025). Functionally, ZBP1’s activation is governed by a complex multi-layered system. This includes physical thresholds for higher-order assembly (Xie et al., 2024), isoform-mediated tuning (Nagata et al., 2024), and inflammation is largely scaffolding-dependent, whereas cell death execution is kinase-dependent (Peng et al., 2022). Viruses counter ZBP1 through stage-specific antagonism aligned with their replication program. For example, poxviruses produce E3 early in replication to limit Z-RNA detection, and herpesviruses express vRHIMs to inhibit ZBP1-RIPK3 interactions (Ali et al., 2019). Accordingly, these interactions span spatial, temporal, and molecular dimensions rather than representing a single linear pathway. However, this complexity also implies context-dependent limitations suggesting that ZBP1 might not function as a universally dominant sensor and its contribution could be redundant or minor depending on the specific viral antagonists and cellular environment. Despite progress, critical unknowns remain. First, relationships between a virus’s replication efficiency and its capacity to induce Z-NAs, or how viruses balance replication speed against immune invisibility. The high replication fidelity of some proofreading-capable viruses, for instance, may represent an evolutionary strategy to minimize the production of Z-NAs and other PAMPs to better evade ZBP1 surveillance (Hsu et al., 2021). Second, the role of ZBP1 in cross-species transmission is another key unknown, especially given the marked species specificity of host-virus co-evolution (Zhang et al., 2023c). When a virus jumps into a new host, its encoded immune antagonists may fail to effectively inhibit the ZBP1 pathway in that species; such mismatches could lead to dysregulated activation of ZBP1 signaling, potentially contributing to cytokine storms (Yang et al., 2025a), which may partially explain the profound pathological injury observed in certain emerging zoonoses (such as COVID-19) in humans.

A greater understanding of the dynamic tussle between ZBP1 and viral replication will ultimately bring forth new concepts and targets for antiviral therapy. The ZBP1 pathway shows context-dependent effects, indicating therapeutic strategies focused on it must take the timing and disease context into cautious consideration. This dual nature drafts a blueprint for precise therapy. For viruses that rely on antagonists to subdue ZBP1, the therapeutic goal is to activate its anti-replication activity. Given the current lack of direct agonists, translational strategies have focused on repurposing agents such as interferons to enhance expression or SMAC mimetics to sensitize downstream signaling, while the disruption of viral antagonists remains a promising theoretical direction. Conversely, in clinical contexts where ZBP1 signaling drives extensive cellular damage and systemic inflammation (Karki et al., 2022), the therapeutic focus must shift to suppressing the pathway. Clinically, this is currently achieved through indirect means, ranging from upstream suppression of ZBP1 expression via JAK inhibitors like Baricitinib to the blockade of downstream effectors such as RIPK1 (Kalil et al., 2021). Recent work has reported covalent PROTACs capable of inducing ZBP1 degradation in cellular models. These findings indicate that, despite prior challenges with direct targeting, pharmacological modulation of ZBP1 activity may become feasible with further development. Ultimately, the future of clinical practice lies in identifying the primary driver of disease. This necessitates determining whether viral replication or host immunopathology is dominant to decide whether activating or, more plausibly, suppressing the ZBP1 pathway will achieve individualized precision therapy.
